# Co-circulation of *Trypanosoma cruzi* and *Leishmania spp*. in Northwestern Colombia, a major migratory corridor

**DOI:** 10.1371/journal.pntd.0014496

**Published:** 2026-07-10

**Authors:** Omar Cantillo-Barraza, Adriana Pabón, Hugo O. Valdivia, Laura Posada, Sofia Machado, Natalia Velásquez-Ortiz, Yurany Granada, Juan F. Sanchez, Omar Triana, Gissella M. Vasquez, Alberto Tobón-Castaño

**Affiliations:** 1 Grupo Biología y Control de Enfermedades Infecciosas, Universidad de Antioquia, Medellín, Colombia; 2 Grupo Malaria, Facultad de Medicina, Universidad de Antioquia, Medellín, Colombia; 3 U.S. Naval Medical Research Unit SOUTH (NAMRU SOUTH), Lima, Perú; 4 Unidad de Eco-epidemiología, PECET, Universidad de Antioquia, Medellín, Colombia; Instituto de Investigaciones Biotecnológicas, ARGENTINA

## Abstract

*Trypanosoma cruzi* and *Leishmania* spp. are the causative agents of Chagas disease and Leishmaniasis, respectively. These flagellates have complex transmission cycles involving a variety of mammal hosts, some of which may serve as potential reservoirs for both parasites. The region of Urabá, located in northwestern Colombia, is the most widely used migratory route in Latin America where the largest migration phenomenon in the last 15 years has occurred. In this study, we evaluated the co-circulation and co-infections with *T. cruzi* and *Leishmania* spp. in the municipalities of Apartadó, Turbo, Necoclí and San Pedro de Urabá using a One Health approach. Integrated efforts included (i) a cross-sectional serological study to assess human infection, (ii) an entomological survey to assess the presence and natural infection of vectors, and (iii) an evaluation of synanthropic mammal infection by both parasites using molecular tools. Our study found recent *T. cruzi* transmission in Turbo and Necoclí, with an infection frequency of 2.42% (95% CI: 1.28–3.56), and identified 11 cases of cutaneous leishmaniasis, with two individuals coinfected with both pathogens. We collected 57 triatomine bugs (*Rhodnius pallescens*) and found that 68.4% (n = 39) were infected with *T. cruzi*. In addition, we collected 2,334 sand flies, with *Pressatia dysponeta* as the most abundant species (79.9%), and detected natural *Leishmania* spp. infection in *Lutzomyia gomezi* (n = 3)*, Psychodopygus panamensis* (n = 1) and *Nyssomyia trapidoi* (n = 1). Finally, *Didelphis marsupialis* (n = 21) was the most frequently captured mammal; 40,9% tested positive for *T. cruzi*, 27,3% for *Leishmania* spp., and one specimen was positive for both pathogens. This study highlights the active transmission and co-circulation of *T. cruzi* and *Leishmania* spp. parasites in Urabá, demonstrating not only the high risk for the local population but also for migrants traversing this area, raising concerns about the potential spread of these parasites to other regions of the continent.

## Author summary

The region of Urabá in northwestern Colombia is a major migratory corridor for people traveling to North America and is an area where neglected tropical diseases such as leishmaniasis and Chagas disease are relatively common.

We conducted a study in different communities in Urabá, collecting samples from humans, triatomine bugs, sand flies, and small mammals for the detection of parasites associated with Chagas disease and leishmaniasis. Our results showed that residents were infected with these parasites, with two of them positive for both diseases. We also found a high percentage of *T.* *cruzi*-positive triatomine bugs (68.4%) and identified three species of sand flies infected with *Leishmania*. Furthermore, the common opossum (*Didelphis marsupialis*) was frequently infected with *T. cruzi*, *Leishmania*, or both, suggesting that it could be an important potential reservoir maintaining these parasites in the environment.

These findings confirm that the conditions in the Urabá region support the active transmission of both Chagas disease and leishmaniasis. This poses a significant health risk to the vulnerable local and migrant populations and underscores the importance of monitoring the dispersal of these pathogens toward new regions, facilitated by high human mobility through this transcontinental corridor. Our study highlights the urgent need for integrated public health strategies that consider human, animal, and environmental factors to control these neglected tropical diseases.

## Introduction

Urabá is a geographical subregion of Colombia located at the confluence of the departments of Antioquia and Chocó at the border with Panamá. The area is named after the Gulf of Urabá, which is located nearby and is recognized for its strategic geographical location as it is a crossroad between the Pacific and Atlantic oceans, the two largest oceans in the global economy, and between North and Central/South America [1]. The Urabá region in Colombia serves as a key transit corridor for mixed migratory flows moving from South to North America [2]. Between 2016 and 2021, authorities documented transit through the Darien Gap involving individuals from 102 different nationalities, primarily from the Americas, Africa, and Asia [2]. Consequently, the border area between Colombia and Panamá acts as a mandatory bottleneck for these diverse groups traveling by land, as they converge in this region due to the lack of alternative air or sea routes [2]. The massive arrival of migrants poses challenges in crucial areas such as security, safety, health, housing, and access to drinking water, food, and basic services.

Urabá is an endemic area for different vector borne diseases (VBD) including Chagas disease and leishmaniasis. Chagas disease (CD) is endemic in Latin America, especially in rural areas where triatomine bugs mediate transmission [3]. The Panamerican Health Organization estimated that up to 100 million people are at risk of infection with 8 million people already infected and 10,000 deaths reported every year [3,4]. CD is also considered a global threat because human migration can expand transmission to non-endemic regions [5]. Leishmaniasis is also a VBD associated with poverty, with an estimate of 700,000–1 million cases per year in approximately 100 endemic countries [6]. The two parasites, *Trypanosoma cruzi* and *Leishmania* spp., are present in a variety of reservoir hosts, both wild and domestic (including humans), which play a key role in the epidemiology of both diseases [7].

Serological and molecular surveillance of humans and other mammals have a key role in enhancing our understanding of the transmission cycles for both parasites [8], as they generate useful data to inform relevant stakeholders and guide preventive measures [9–11]. Due to the complexity of transmission cycles of CD and leishmaniasis, an integrative “One Health” [12] approach is the most effective strategy for control and prevention because it involves relevant aspects of hosts, vectors, parasites and environment [13,14].

It has been previously reported that human migration contributes to the spread of diseases from endemic to non-endemic regions, including the United States and Europe as primary destinations for migrant populations [15]. The role of migration in the spread of disease should not be underestimated. The Venezuelan humanitarian crisis, which has driven millions of people to migrate across Central and South America, raises concerns about the spread of Neglected Tropical Diseases around the region [14,16].

Historically, the Urabá-Antioquia area has had the most reports of leishmaniasis cases in Colombia, and a wide diversity of sandflies and CD relevant triatomines, particularly *Rhodnius pallescens* and *Triatoma dimidiata* with the former highly associated with palm tree ecotopes, as well as *Panstrongylus geniculatus*, which does not typically live indoors but has been reported entering homes, and finally the indoor *Rhodnius prolixus*, which was introduced and has not been reported in the area since the chemical control measures were implemented [17]. However, there are no studies regarding co-circulation of both pathogens, risk of infection among resident populations and the role of animal reservoirs in disease transmission and persistence in this region [18]. Therefore, we conducted a One Health surveillance study of *T. cruzi* and *Leishmania* spp. in four municipalities (Apartadó, Turbo, Necoclí, and San Pedro de Urabá) in the Urabá region to shed light into the dynamics occurrence of co-infection of these pathogens in this key migratory hub in the continent.

## Methodology

### Ethics statement

#### Ethics statement for human and animal research.

This study was reviewed and approved by the Institutional Review Board (approval #20-32-924 of 2020) and the Institutional Animal Care and Use Committee (approval #138 of 2021) of the Universidad de Antioquia. All adult participants (aged 18 years or older) signed the informed consent form whereas children (under 18 years old) were enrolled after their parents or guardians signed the informed consent form on their behalf. All animals were handled in strict accordance with the Colombian Code of Practice for the Care and Use of Animals for Scientific Purposes, as established by Law 84 of 1989, to ensure good animal welfare. The protocol for this study (NAMRU6.2018.0002) was reviewed and approved by the Research Administration Program of the U.S. Naval Medical Research Unit SOUTH.

### Study area

**Fig 1:**
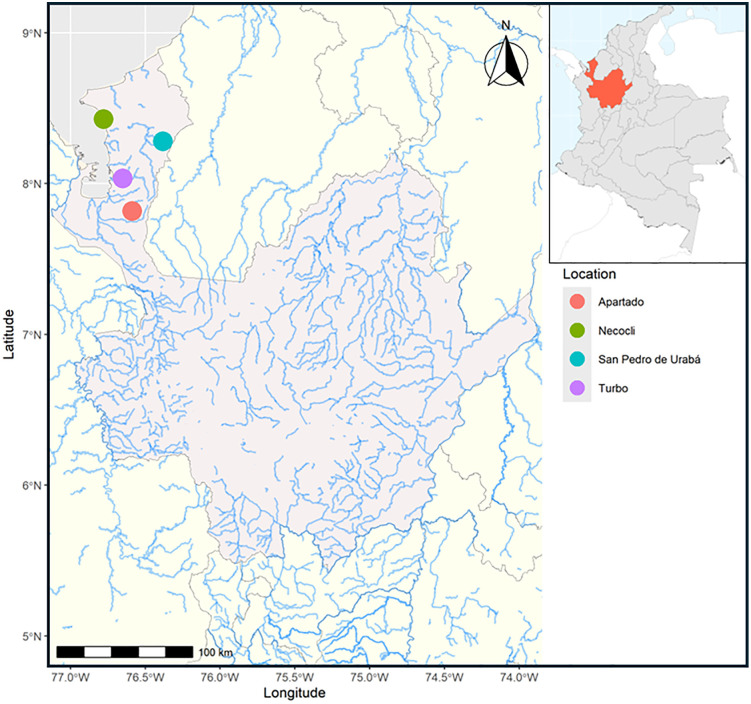
Map of study sites in the Urabá region, Antioquia, Colombia. Map created in R using open data from the GADM database of Global Administrative Areas, version 3.6. URL: www.gadm.org.

This descriptive study was conducted from October 2021 to October 2024 in four municipalities in the Urabá region (Fig 1), Department of Antioquia. Apartadó (7.8833° N, 76.6333° W) is the most populous municipality in Urabá and is located at 25 meters above sea level (m.a.s.l.) with temperatures between 24 to 32°C; Turbo (8.0650° N, 76.7361° W) is the largest municipality of Antioquia by area, with 57% of its inhabitants living in rural areas, and located at 2 m.a.s.l. with an average temperature of 28°C. Necoclí (8.2197° N, 76.7211° W) is located on the eastern shore of the Gulf of Urabá (Caribbean Sea) at 6 m.a.s.l. and has an average temperature of 28°C; and San Pedro de Urabá (7.7819° N, 76.5394° W) is located at 200 m.a.s.l., with an average temperature of 27°C and more than half of its inhabitants (59.1%) living in rural areas.

### Entomological sampling

Insects were collected from February 2022 to February 2024 in urban and rural areas of each municipality. For triatomine collections, we checked the leaves, bracts and organic debris of *Attalea butyracea* palm trees located less than one kilometer from nearby households (peridomestic areas) following a previously reported methodology [19]. Additionally, during the blood sampling activities, the research team displayed entomological specimens of triatomine species identified in the area to community members, aiming to promote their engagement in the collection of reduviid bugs within their households. Collected triatomine bugs were taken to the Biología y Control de Enfermedades Infecciosas (BCEI) laboratory at Universidad de Antioquia where they were identified using taxonomic keys [20].

Sand fly collections were performed during three consecutive nights at each location using six CDC automatic light traps deployed in peridomestic areas from 6:00 PM to 6:00 AM. Collected sand flies were stored dry in tubes for subsequent identification and analysis. Specimens were identified using taxonomic keys and procedures described by Galati [21], and generic abbreviations were applied according to Marcondes [22].

### Human sample collection and epidemiological survey

Community leaders in urban and rural areas of each municipality were contacted in advance to inform them about the study objectives, methodology and relevance. Surveillance was prioritized in rural locations, populated centers and scattered areas following recommendations from health authorities. Participants were recruited between February 2023 to February 2024 through active search methodology in different locations in the community such as households, local health centers, or hospitals. After obtaining written consent from each participant, we collected epidemiological data and 5 mL of blood was centrifuged and the serum was preserved under cold chain until testing. Regarding leishmaniasis study, all participants in the serological study were asked about the presence of lesions consistent with leishmaniasis. All subjects with skin lesions were invited to participate in the study. We excluded participants with a previous parasitological diagnosis, clinical evidence of bacteria or fungi, or facial or genital lesions. We collected skin biopsies from the lesions using a sterile 3-mm punch. Samples were stored in molecular-grade alcohol and preserved at room temperature prior to testing.

### Synanthropic mammals sampling

Following the methodology of Quintero *et al* 2022, developed in Urabá [23] and Cantillo *et al* 2015 in the Caribbean region [24], synanthropic mammals were captured using Tomahawk and Sherman live traps baited with a mixture of peanuts, banana, oat, and fish. At each locality, 10 traps were set for three nights in peridomestic areas and in the forests where palms were sampled, and were distributed along linear transects with capture points established every 20 m. To detect *T. cruzi*, trapped mammals were anesthetized intramuscularly (9:1 ketamine hydrochloride 10%, and xylazine 2%), according to Roque &Jansen, 2014 [25]. Blood samples were stored under a cold chain for DNA extraction and molecular diagnosis. For *Leishmania spp.* detection, ear biopsies were performed on each animal, using the same sampling methodology described for human cases.

### Molecular detection of *T. cruzi* and *Leishmania* spp. in insects, animals and humans

DNA from triatomine bugs and mammals’ blood was extracted using the Invisorb Spin Universal Kit (STRATEC Molecular GmbH), following the manufacturer’s instructions. *Trypanosoma cruzi* was detected by conventional PCR targeting the satellite DNA using primers cruzi1 (5′-AST CGG CTG ATC GTT TTC-3′) and cruzi2 (5′-AAT TCC TCC AAG CAG CGG ATA-3′), according to the protocol from Hernández and colleagues [26]. Positive *T. cruzi* samples were analyzed for molecular discrimination of TcI Discrete Typing Unit (DTU) from other DTUs based on the amplification of the spliced leader intergenic region (SL-IR) gene using primers TCC (5′-CCC CCC TCC CAG GCC ACA CTG 3′), TC1 (5′GTG TCC GCC ACC TCC TTCGGG CC-3′) and TC2 (5′-CCT GCA GGC ACA CGT GTG TGT G-3′) [27]. Amplification products were run on a 1.5% agarose gel, stained by ethidium bromide, and visualized under UV light.

For detection of *Leishmania* infection, a subset of collected female sand flies was dissected, with the head and last abdominal segments used for morphological species identification, while the thorax and anterior abdominal segments were preserved dry at -20°C for molecular analyses. DNA was extracted from sand flies, mammal and human biopsies using the QIAamp DNA blood Mini kit (QIAGEN) following the manufacturer’s instructions. *Leishmania* DNA was detected by conventional PCR that amplifies a 115-bp product of the 18S rRNA gene using the primers 18S-L-F5′-CGTAGTTGAACTGTGGGCTGTGC- 3′ and 18S-L-R 5′-ACTCCCGTGTTTCTTGTTTC TTTGAA-3′ as previously described [28]. PCR products were run on a 2% agarose gel and visualized in the gel documentation.

### *Trypanosoma cruzi* serology in humans

Two serological tests based on different principles were performed to detect anti-*T. cruzi* IgG, following the recommendations of the National Institute of Health, Colombia. All samples were first screened using a total antigen ELISA (ELISA Chagatest Wiener). Samples that tested positive by ELISA were subsequently confirmed using a recombinant ELISA (CHAGATEK ELISA, MicroELISA system) for *T. cruzi*, according to the manufacturer’s instructions. Only samples positive in both assays were considered truly seropositive.

## Results

### Seropositivity of *T. cruzi* infection in humans and Leishmaniasis evidence

A total of 702 human serum samples were collected from participants aged 1–90 years, including 430 women (61.2%) and 272 men (38.8%) (Table 1). Most study participants (75.8%) were either housewives or farmers and Turbo accounted for 38% of all subjects. Nearly half of the population reported headaches and a third of the population reported fever at the time of enrolment.

**Table 1:** Characteristics of participants by serologic status for Chagas disease.

Characteristics	Serostatus for Chagas disease		p-value
**Seronegative (n = 688)** **n (%)**	**Seropositive (n = 14)** **n (%)**
**Socio-demographic**			
Age (years)*	38.4 ± 20.5	39.1 ± 19.4	0.907
sex			
Male	258 (37.6)	10 (71.4)	**0.010**
Female	428 (62.4)	4 (28.6)	
Occupation (n = 512)**			
Housewife	249 (49.8)	4 (33.3)	**0.047**
Farmer	130 (26.0)	5 (41.7)	
Student	81 (16.2)	0 (0.0)	
Other occupation	40 (8.0)	3 (25.0)	
Communities**			
Turbo	264 (38.4)	3 (21.4)	0.355
Necocli	182 (26.4)	4 (28.6)	
San Pedro	170 (24.7)	4/28.6)	
Apartado	72 (10.5)	3 (21.4)	
**Clinical characteristics**			
Current symptoms (n = 639)			
Fever**	159 (28.2)	2 (16.7)	0.525
Headache	321 (51.4)	4 (28.6)	0.092
Malaise**	204 (33.3)	1 (7.1)	**0.043**
Chills**	138 (22.6)	2 (14.3)	0.746
Sweating**	79 (12.9)	0 (0.0)	0.236
Pallor**	30 (4.9)	1 (7.1)	0.513
Anorexia**	29 (4.8)	0 (0.0)	1.000
Joint pain	264 (43.1)	5 (35.7)	0.579
Muscle pain	247 (40.2)	3 (21.4)	0.157
**Epidemiological**			
Travel during the last 3 months*	99 (19.7)	1 (8.3)	0.476
Recent use of insecticide	99 (19.7)	1 (8.3)	0.325

* Mean ± Standard Deviation.

** Fisher’s Exact Test.

Serological screening detected 14 individuals who tested positive for *T. cruzi* infection. Out of those, four were from Necoclí, four from San Pedro de Urabá, three from Turbo, and three from Apartadó (Table 1). This resulted in an overall infection rate of 1.99% (95% CI: 0.96-3.32) (Table 1). Interestingly, a 12-year-old participant of Turbo and a 7-year-old in Necoclí were seropositive. Bivariate analysis identify differences in sex, occupation and malaise between seronegative and seropositive cases (Table 1). Additionally, 12 individuals with suspected cutaneous leishmaniasis lesions were evaluated, and *Leishmania* DNA was detected in 91.7% (11/12) of them. Two individuals (16.7%), aged 21 and 36, from Turbo and San Pedro de Urabá, respectively, were found to be co-infected with both *T. cruzi* and *Leishmania* spp.

### *Trypanosoma cruzi* natural infection rate in triatomines

**Fig 2:**
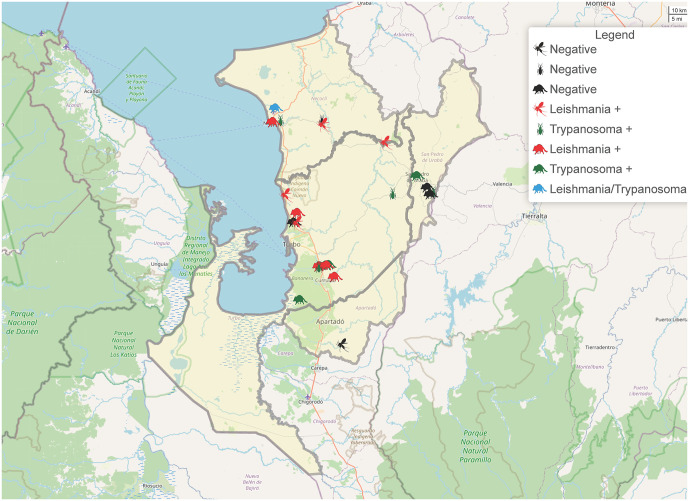
Geographic distribution of triatomines and mammals and their infection status. Symbols represent the type of specimen (insect or mammal), while colors indicate the diagnostic result: black for negative samples, red for *Leishmania* spp., green for *Trypanosoma* spp., and blue for mixed infections (co-infection). Map created in R using open data from OpenStreetMap (https://www.openstreetmap.org/).

A total of 57 triatomine bugs, all identified as *Rhodnius pallescens,* were collected in palms in three of the four municipalities studied (Fig 2). No kissing bugs were submitted by the community. All specimens were evaluated for *T. cruzi.* All *R. pallescens* collected in San Pedro de Urabá (14/14) were positive for *T. cruzi*; 68.5% (11/16) in Necoclí and 51.8% (14/27) in Turbo (Table 2). Only TcI was found in positive samples.

**Table 2:** Summary of *T. cruzi* and *Leishmania* spp. infection in humans, vectors, and synanthropic mammals in four municipalities of Urabá, Colombia. *P. semispinosus* and *Sylvilagus* sp. are not listed since only one specimen was collected for each and were negative for *T. cruzi* and *Leishmania.*

Category	Measure	Apartadó	Turbo	Necoclí	San Pedro de Urabá	Total
Humans	Screened	75	267	186	174	702
	*T. cruzi* + (%)	3 (4.0%)	3 (1.1%)	4 (2.2%)	4 (2.3%)	14 (2.0%)
	*Leishmania* + (%)	0	2 (0.8%)	8 (4.3%)	1 (0.6%)	11 (1.6%)
Triatomines	R. pallescens	0	27	16	14	57
	*T. cruzi* + (%)	N/A	14 (51.8%)	11 (68.5%)	14 (100%)	39 (68.4%)
Sand flies	Collected	6	2134	145	58	2334
	Leishmania +	0	3*	2**	0	5
Mammals	*D. marsupialis*	0	11	7	3	21
	*T. cruzi* + (%)	N/A	5 (45.5%)	2 (28.6%)	1 (33.3%)	8 (38.1%)
	*Leishmania* + (%)	N/A	2 (18.2%)	3 (42.9%)	0 (0%)	5 (22.7%)
	Co-infection (%)	N/A	0 (0%)	1 (14.3%)	0 (0%)	1 (4.5%)

* All positives found in *Lutzomyia gomezi.*

** One positive was found in *Psychodopygus panamensis* and in *Nyssomyia trapidoi*.

N/A (Not Applicable): no specimens of this category were collected in this municipality

### Sand flies and natural infection with *Leishmania* spp

A total of 2,334 sand flies were collected (1,188 females and 1,146 males), representing 13 species across eight genera (S1 Table). The most abundant species were *Pressatia dysponeta* (79.9%), *Lutzomyia gomezi* (8.8%) and *Psychodopygus panamensis* (8.1%). Among 58 females of the species *Lu. gomezi*, *Ps. panamensis,* and *Nyssomyia trapidoi*, 5 were leishmania-positive (3 *Lu. gomezi,* 1 *Ps. panamensis*, and 1 *Ny. trapidoi*)*.*

### Trypanosoma cruzi and Leishmania spp. infection in synanthropic mammals

We captured 23 mammals, with *Didelphis marsupialis* as the most abundant species (21 individuals), followed by *Proechymis semispinosus* and *Sylvilagus* sp*.* (1 individual each)*.* Regarding *Didelphis marsupialis.* Screening showed positive samples on *Didelphis marsupialis* with 9 specimens (43%) positive only for *T. cruzi*, 5 (24%) only for *Leishmania*, and 1 (5%) showed co-infection with both pathogens (Table 2). All *T. cruzi* positives were infected with TcI. No infection was detected in the other mammals. Regarding the geographic distribution of *D. marsupialis*, 11 individuals were collected in Turbo, with 5 (45.5%) infected with *T. cruzi* and 2 (18.2%) with *Leishmania* spp. In Necoclí, 7 individuals were captured; among them, 2 (28.6%) were infected only with *T. cruzi*, 3 (42.9%) only with *Leishmania* spp., and 1 (14.3%) with both pathogens. Finally, 3 individuals were collected in San Pedro de Urabá, with 1 (33%) testing positive for *T. cruzi* and no evidence of *Leishmania* spp. infection.

## Discussion

The Urabá region in northwestern Colombia has undergone significant human-driven changes due to agricultural development, large-scale human migration, and high levels of poverty and social inequality [29]. Although these factors support the persistence of VBD such as leishmaniasis and CD, research on co-circulation and co-infection between *T. cruzi* and *Leishmania* spp*.* in humans and mammals has not yet been conducted. Our study offers the first comprehensive eco-epidemiological study of active transmission and coinfection by *Trypanosoma cruzi* and *Leishmania* spp. in this strategic area through a One Health approach in humans, vectors, and synanthropic reservoirs.

The detection of *T. cruzi* transmission in Turbo, along with the presence of infected *R. pallescens* and *D. marsupialis* in the same locality, suggests that non-domiciliated triatomines associated with *Attalea butyracea* palms may have an increasing role in transmission in this region, as reported previously in the Caribbean region [24]. Furthermore, the lack of primary CD vectors at our study sites suggests that *T. cruzi* transmission may be related to the intrusion of secondary triatomines like *R. pallescens* into homes [30–32] as reported in the nearby town of San Juan de Urabá [33] and other areas of the Colombian Caribbean [29,32]. The enzootic *T. cruzi* transmission cycle close to the resident population is further supported by high infection rates found in *R. pallescens* (68.5%) and *D. marsupialis* (39.1%), which coexist in the palm groves surrounding the study areas. We suggest that recent transmission may be occurring in this area, probably associated with the enzootic cycle, given the presence of seropositive children in Necoclí and Turbo. The seropositive children aged 7 and 12 years, were confirmed to have been born and to have lived their entire lives in Necoclí and Turbo, respectively, supporting the likelihood of local transmission. For chronic infections such as Chagas disease, the presence of *T. cruzi* infection in children and young individuals suggests recent or ongoing transmission, while infection in adults may represent exposure acquired in the past [34–36]. However, congenital transmission cannot be excluded, as the mothers of the seropositive children were not evaluated.

The three sand fly species found infected with *Leishmania* spp. (*Lu. gomezi*, *Ps. panamensis* and *Ny. trapidoi*) are epidemiologically relevant for leishmaniasis transmission [37]. A previous study from the neighboring municipality of Carepa [38] suggested that the coexistence of *Ps. panamensis* and *Ny. trapidoi* could imply greater complexity in the transmission cycles of leishmaniasis since *Ny. trapidoi* (secondary vector) could replace *Ps.* *panamensis* as primary vector in its absence [38]. The presence of these species in the study area is significant, as *Ny. trapidoi* is a vector of *L. braziliensis, L. panamensis* and *L. mexicana* in the Pacific region, while *Ps. panamensis* is a vector of *L. braziliensis* and *L. panamensis* in the valley of Magdalena River and Darién region. Their occurrence in Urabá reinforces the potential risk of cutaneous leishmaniasis transmission in the area [37,39].

In terms of animal potential reservoirs, the detection of high rates of *D. marsupialis* positive to *T. cruzi* suggests a risk for human transmission in Turbo, Necoclí and San Pedro de Urabá. This species is widely distributed from Central to South America, has relevance in human and veterinarian health [40] and may act as an important peridomestic *T. cruzi* reservoir, being associated with oral outbreaks and reported to triple the risk of Chagas disease transmission [26,30,41]. This species is also considered a potential reservoir for *L. infantum* and at least four other species of *Leishmania* in the Caribbean coast and Pacific region [42–48]. In this regard, our data also showed *Leishmania* spp. infection in this species in Necoclí (42.9%) and Turbo (18.2%).

Unlike reports from neighboring countries such as Venezuela and Brazil, there is little data from Colombia regarding coinfection of this mammal with *T. cruzi* and *Leishmania* spp. [45,46]. The data presented here support potential coinfection in humans and *D. marsupialis* in our study locations with the presence of infected vectors (*R. pallescens*, *Lu. gomezi,* and *Ps. panamensis*) and recent transmission of both parasites.

Besides the risk for the local population in Urabá, our data also suggests a risk for the transit population [49,50]. The highest historical numbers of migrants entering Colombia have been reported in the last 15 years, primarily Venezuelan migrants. There was a first peak in 2018 with 2,665,959 migrants [51], followed by a second peak in 2022 with 2,896,748 migrants [2]. Additionally, a new migration pattern has emerged, with many migrants now considering Colombia their final destination.

The massive urban spread could shift the epidemiology of these diseases as a result of migrants’ barriers to adequate healthcare and living conditions, which leave them with untreated infections that can spread to their main destination within Colombia, Europe or the United States [14,50]. One example illustrating this change for CD is the detection of DTUs specific to other endemic regions, as shown by reports of TcV and TcVI hybrids in an area in Colombia with no natural reservoirs for these DTUs, supporting an anthropogenic introduction [52].

This situation poses critical challenges for the control and surveillance of infectious diseases, as migrants could become infected by passing through endemic zones, increasing the incidence of these diseases in countries where they were not present before. Strategies to address these challenges contemplate a One Health approach, articulating human, animal and environmental health within a multidisciplinary framework, and molecular tools that determine whether strains are native or imported [53].

This study has several limitations that should be considered when interpreting the results. First, the cross-sectional study design limits any assessment of transmission dynamics over time. Also, the molecular characterization of *Leishmania* species was not performed, preventing us to elucidate the potential distribution of *Leishmania* species circulating in humans, vectors, and mammalian hosts. Finally, sampling could not be conducted during both dry and rainy seasons because of security concerns in the study areas, which limited the evaluation of potential seasonal variations.

Nevertheless, our results show that Urabá has conditions that promote the transmission of *T. cruzi* and *Leishmania* spp., posing a risk to local and migrant populations and threatening the spread of these diseases to other regions. Transmission in this area seems to be driven via non-domiciliated triatomines as well as primary and secondary sand fly vectors and *D. marsupialis* as a key wild reservoir for both diseases.

Educating the population to minimize direct or indirect contact with this animal is likely the best strategy under a One Health context as it takes into consideration the environment as well as human and animal health [40]. In this context, new strategies should explore (i) active community participation in entomological surveillance, (ii) identification of natural ecotopes, dispersal patterns, and feeding sources of key vectors, and (iii) the implementation of preventive measures in peridomestic areas, such as the ecological management of *A. butyracea* palm trees to reduce vector colonization near dwellings.

## Supporting information

S1 Table Collected sand flies using CDC light traps at each community. (XLSX)
